# Integrated Assessment of Bioactive Properties of Nine *Thlaspi* Species: Antioxidant Activity, Enzyme Inhibition and LC–MS/MS Polyphenolic Characterization

**DOI:** 10.3390/plants15142207

**Published:** 2026-07-20

**Authors:** Hasan Karageçili, Zeynebe Bingöl, Mustafa Abdullah Yılmaz, Mehmet Fidan, Mehmet Cengiz Karaismailoğlu, Hulya Akıncıoglu, İlhami Gulcin

**Affiliations:** 1 Department of Nursing, Faculty of Health Sciences, Siirt University, 56100 Siirt, Türkiye; 2 Department of Medical Services and Techniques, Tokat Vocational School of Health Services, Gaziosmanpasa University, 60250 Tokat, Türkiye; zeynebe.bingol@gop.edu.tr; 3 Faculty of Pharmacy, Department of Analytical Chemistry, Dicle University, 21280 Diyarbakır, Türkiye; mustafaabdullahyilmaz@gmail.com; 4 Department of Biology, Faculty of Science and Arts, Siirt University, 56100 Siirt, Türkiye; mfidan7384@hotmail.com; 5 Department of Molecular Biology and Genetics, Faculty of Sciences, Bartın University, 74100 Siirt, Türkiye; mkaraismailoglu@bartin.edu.tr; 6 Faculty of Pharmacy, Department of Biochemistry, Agri Ibrahim Cecen University, 04100 Agri, Türkiye; hgakincioglu@agri.edu.tr; 7 Department of Chemistry, Faculty of Science, Ataturk University, 25240 Erzurum, Türkiye; 8 Rectorate of Agri Ibrahim Cecen University, 04100 Agri, Türkiye

**Keywords:** *Thlaspi*, Brassicaceae, antioxidant activity, α-amylase, acetylcholinesterase, carbonic anhydrase, phenolic compounds

## Abstract

Brassicaceae plants, among the most widely consumed vegetables worldwide, are recognized as rich sources of biologically active compounds. In this study, nine species belonging to the cruciferous genus *Thlaspi* were investigated, including *T. alliaceum*, *T. arvense*, *T. violascens*, *T. aghricum*, *T. cataonicum*, *T. annuum*, *T. watsonii*, *T. cariense*, and *T. elegans*. In the past, pennycress species were consumed to alleviate hunger and provide nutritional support during periods of food scarcity. To evaluate the antioxidant capacities of methanol and water extracts obtained from *Thlaspi* species, several complementary assays were employed, including 2,2′-azino-bis-3-ethylbenzthiazoline-6-sulphonic acid radical (ABTS^•+^) scavenging, 1,1-diphenyl-2-picrylhydrazyl free radicals (DPPH^•^) scavenging, N,N-dimethylphenylenediamine radicals (DMPD^•+^) scavenging, Fe^3+^-2,4,6-tris(2-pyridyl)-s-triazine (TPTZ)-reducing, Fe^3+^ ion-reducing, and Cu^2+^ ion-reducing assays. The IC_50_ values of both methanol and water extracts from the aerial parts of *Thlaspi* species for ABTS^•+^, DPPH^•^, and DMPD^•+^ scavenging activities were studied compared with antioxidant standards, including α-tocopherol, Trolox, butylated hydroxytoluene (BHA), and butylated hydroxyanisole (BHT). The total phenolic and flavonoid contents of the extracts ranged from 8.29 to 49.14 mg gallic acid equivalent (GAE)/g extract and from 2.33 to 74.66 mg quercetin equivalent (QE)/g extract, respectively. Furthermore, the inhibitory effects of water and methanol extracts of *Thlaspi* species against α-amylase, acetylcholinesterase (AChE), and carbonic anhydrase (CA II) enzymes were evaluated. The IC_50_ values were determined to range from 122.4 to 245.9 μg/mL for α-amylase, from 17.3 to 24.1 μg/mL for AChE, and from 41.9 to 256.5 μg/mL for CA II inhibition. In addition, the phenolic profiles of *Thlaspi* species were comprehensively characterized by LC-MS/MS analysis using 53 reference standards. The findings demonstrated that the aerial parts of *Thlaspi* species are rich in polyphenolic antioxidants and may serve as promising natural sources with potential applications in the management of diabetes, Alzheimer’s disease (AD), glaucoma, epilepsy, and cancer.

## 1. Introduction

Plants belonging to the Brassicaceae family are among the most widely consumed vegetables worldwide and are recognized as rich sources of bioactive phytochemicals [1]. Members of this family are particularly abundant in polyphenols, flavonoids, and anthocyanins, which contribute significantly to their nutritional and medicinal value. In addition, hydroxycinnamic acids such as ferulic, sinapic, and p-coumaric acids are frequently detected, often conjugated with sugars or other phenolic acids [2]. Among the genera of Brassicaceae, *Thlaspi* represents one of the largest and most diverse groups, comprising approximately 75 species distributed mainly throughout Eurasia. Turkey is considered an important center of diversity for the genus, hosting 36 taxa at different taxonomic levels. Remarkably, 21 of these taxa are endemic to Turkey, corresponding to an endemism rate of approximately 58% [3,4]. The genus *Thlaspi* has attracted increasing attention because of its ecological, industrial, and ethnobotanical importance. Certain species have been utilized in ornamental cultivation, biodiesel production, and phytoremediation due to their ability to accumulate heavy metals from contaminated soils [5]. Historically, pennycress was consumed during periods of food scarcity to suppress hunger and provide nutritional support [6]. Recent studies have indicated that *T. arvense* contains various biologically active compounds, particularly flavonoids and organic acids, which may exhibit anti-inflammatory and antioxidant effects against hyperuricemia-induced oxidative stress [7]. In recent years, biologically active secondary metabolites obtained from medicinal plants have gained considerable scientific and pharmaceutical interest because of their potential applications in the prevention and treatment of various diseases. Consequently, there has been a growing demand for the identification, characterization, and pharmacological evaluation of natural products derived from plant species such as *Thlaspi* [8].

Reactive oxygen species (ROS) are highly reactive molecules and intermediates derived from molecular oxygen (O_2_). These species are generated through various endogenous and exogenous processes, including mitochondrial electron transport pathways, metal-catalyzed reactions, inflammatory responses mediated by neutrophils and macrophages, environmental pollutants, and exposure to ionizing and ultraviolet radiation such as gamma, X-, and UV-rays [9]. Under physiological conditions, living organisms maintain a delicate balance between ROS generation and antioxidant defense systems. However, excessive ROS production or insufficient antioxidant protection disrupts this balance, leading to oxidative stress [10]. Oxidative stress causes structural and functional damage to essential biomolecules, including lipids, proteins, carbohydrates, and nucleic acids. Such oxidative modifications are closely associated with the pathogenesis of numerous chronic and degenerative diseases, including cancer, cardiovascular disorders, diabetes, and neurodegenerative diseases [11]. To counteract these harmful effects, living organisms utilize complex antioxidant defense systems consisting of antioxidant enzymes and non-enzymatic antioxidant compounds. Antioxidants can delay, inhibit, or prevent oxidative damage by neutralizing free radicals and suppressing oxidation reactions [12].

Plant-derived phenolic and polyphenolic compounds are among the most effective natural antioxidants because of their strong radical scavenging and reducing capacities. Therefore, antioxidant-rich foods and dietary supplements may help reduce oxidative damage induced by ROS and other free radicals [13]. Numerous natural antioxidants isolated from plants have been identified as efficient scavengers of reactive oxygen species and free radicals. In recent years, growing concerns regarding the potential toxic and carcinogenic effects of synthetic antioxidants have increased scientific interest in identifying safer natural alternatives for food, pharmaceutical, and nutraceutical applications [14,15,16,17]. Also, plant-derived antioxidants have gained increasing importance as safer alternatives to synthetic antioxidants, particularly because of concerns regarding the potential adverse effects associated with synthetic compounds [18,19,20].

Alzheimer’s disease (AD) is currently one of the most serious global neurodegenerative disorders and represents a major public health challenge worldwide. Several therapeutic approaches have been explored to manage AD, among which inhibition of acetylcholinesterase (AChE) and butyrylcholinesterase (BChE) enzymes remains one of the most widely accepted strategies for AD treatment [21]. Oxidative stress and lipid peroxidation are strongly implicated in the pathogenesis of AD and other neurodegenerative disorders. Therefore, compounds possessing both antioxidant and cholinesterase (ChE) inhibitory effects are considered promising candidates for neuroprotective therapy [22].

Currently available ChE inhibitors, including tacrine, galantamine, rivastigmine, and donepezil, are clinically used to alleviate AD symptoms. However, their therapeutic application is often limited because of undesirable side effects such as hepatotoxicity and gastrointestinal complications [23]. Early manifestations of AD include progressive memory loss and cognitive impairment, which are closely associated with oxidative stress, neuroinflammation, and acetylcholine (ACh) deficiency [24]. For this reason, antioxidant-rich plant-derived compounds have attracted increasing attention as potential neuroprotective agents capable of slowing the progression of AD and related neurodegenerative disorders [25]. Naturally occurring phenolic compounds have been reported to exhibit significant AChE inhibitory effects and are therefore considered promising bioactive molecules for the development of safer anti-AD therapeutic agents [26].

Type 2 diabetes mellitus (T2DM), accounting for approximately 90–95% of all diabetes cases worldwide, is one of the most prevalent metabolic disorders. The pathogenesis of T2DM involves impaired insulin secretion, insulin resistance, dysregulated lipid metabolism, and abnormal glucose homeostasis [27]. Elevated postprandial blood glucose levels primarily result from the enzymatic digestion of dietary carbohydrates into absorbable monosaccharides. Therefore, inhibition of carbohydrate-hydrolyzing enzymes such as α-amylase and α-glycosidase is considered an effective therapeutic strategy for controlling postprandial hyperglycemia [28].

Carbonic anhydrases (CAs) are zinc-containing metalloenzymes that catalyze the reversible hydration of carbon dioxide (CO_2_) and water into bicarbonate (HCO_3_^−^) and protons (H^+^) [29,30]. These enzymes are involved in numerous physiological and metabolic processes, including acid–base balance, gluconeogenesis, lipogenesis, ureagenesis, and fluid secretion [31,32]. CAs inhibitors (CAIs) have important clinical applications in the treatment of some diseases, including glaucoma, epilepsy, obesity, edema, and certain cancers. Especially, CA II isoenzyme inhibition is an established therapeutic approach for lowering increased intraocular pressure (IOP) associated with glaucoma [33,34,35].

The genus *Thlaspi*, belonging to the Brassicaceae family, has attracted increasing scientific interest because of its ecological importance and rich phytochemical composition. Previous studies have demonstrated that several *Thlaspi* species contain phenolic acids, flavonoids, and other biologically active secondary metabolites that may contribute to antioxidants and enzyme inhibitory activities [3,4]. However, despite the growing interest in medicinal plants as potential natural therapeutic agents, comprehensive comparative studies evaluating the phytochemical composition and biological activities of various *Thlaspi* species remain limited. Specifically, detailed information regarding their antioxidant potential, α-amylase, AChE, and CA inhibitory properties is still insufficient. In this context, the present study aims to comprehensively investigate the total phenolic and flavonoid contents, antioxidant properties, and enzyme inhibitory potentials of methanol and water extracts prepared from the aerial parts of *Thlaspi* species including *T. alliaceum*, *T. arvense*, *T. violascens*, *T. aghricum*, *T. cataonicum*, *T. annuum*, *T. watsonii*, *T. cariense* and *T. elegans*.

## 2. Results

### 2.1. Extraction of Yields and Total Phenolic/Flavonoid Contents

The extraction amounts (g) and yields (%) varied considerably among the *Thlaspi* species investigated and according to the extraction solvent used. Water extraction generally resulted in higher extract yields than methanol extraction. The highest water extraction yield was obtained from *T. arvense* (15.6%), followed by *T. elegans* (13.5%) and *T. cariense* (13.2%), whereas the lowest yield was observed for *T. alliaceum* (5.8%). For methanol extracts, yields ranged from 0.5% to 6.4%, with the highest value recorded for *T. cataonicum* (6.4%) and the lowest for *T. aghricum* (0.5%). These results indicate substantial interspecific differences in extractable constituents and solvent-dependent extraction efficiency (Table 1).

The total phenolic and flavonoid contents of the methanol and water extracts obtained from nine *Thlaspi* species were determined spectrophotometrically and expressed as gallic acid equivalent (GAE) and quercetin equivalent (QE), respectively. The total phenolics ranged between 8.29 and 49.14 mg GAE/g extract, whereas total flavonoid content varied from 2.33 to 74.66 mg QE/g extract. In general, methanol extracts exhibited higher phenolic and flavonoid content compared with water extracts, indicating the stronger extraction efficiency of methanol for polyphenolic compounds. These findings indicate that *Thlaspi* species are rich sources of phenolic and flavonoid compounds, which are known to contribute significantly to antioxidant activity. Previous studies have also reported considerable phenolic and flavonoid content in *Thlaspi* species. In a study investigating Chinese medicinal herbs, the total phenolic and flavonoid contents of the stem and leaf extracts of *Thlaspi arvense* were reported as 41.58 ± 1.68 mg gallic acid equivalent (GAE)/g dried weight (DW) and 2.95 ± 0.38 mg catechin equivalent (CE)/g DW, respectively [36]. Similarly, aqueous and methanol extracts of *T. arvense* collected from Bhutan were evaluated for the first time with respect to their phenolic composition and antioxidant potential. In that study, the aqueous extract contained 5.17 ± 0.25 μg GAE/mg extract of total phenolics and 17.00 ± 0.23 μg quercetin equivalent (QE)/mg extract of total flavonoids, whereas the methanol extract contained 32.64 ± 0.43 μg GAE/mg extract of total phenolics and 59.39 ± 2.99 μg QE/mg extract of total flavonoids [37]. Consistent with these previous reports, the present findings corroborate the finding that *Thlaspi* species possess substantial amounts of phenolic and flavonoid constituents. The detailed total phenolic and flavonoid contents of the *Thlaspi* extracts are presented in Table 1.

### 2.2. LC-MS/MS Profiling

**Figure 1:**
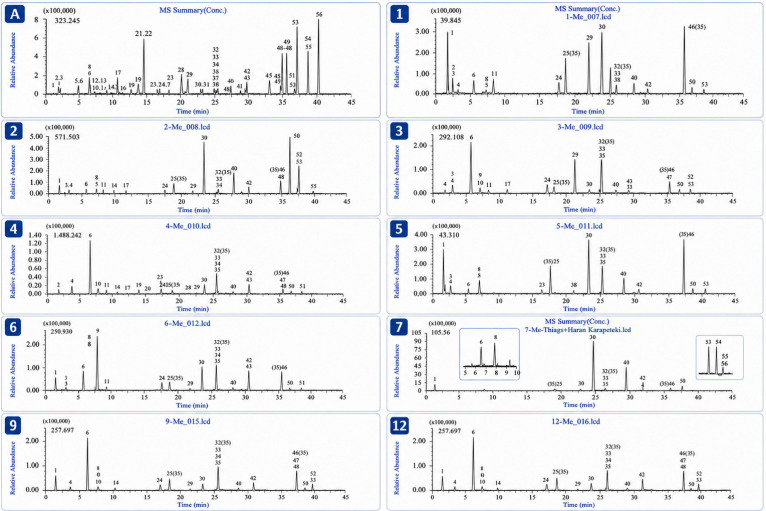
A. LC-MS/MS chromatogram displaying all the standard phenolic compounds that were analyzed. 1: *T. alliaceum* L., 2: *T. arvense*, 3: *T. violascens*, 4: *T. aghricum*, 5: *T. cataonicum*, 6: *T. annuum*, 7: *T. watsonii*, 9: *T. cariense* and 12: *T. elegans* chromatograms of methanol extracts.

**Figure 2:**
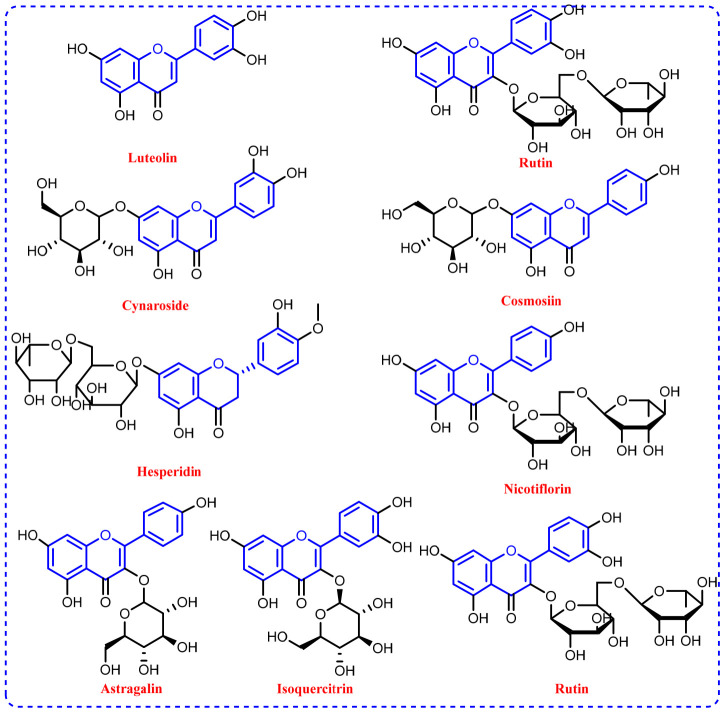
The chemical structure of the ten most abundant phenolic compounds in *Thlaspi* species.

LC–MS/MS parameters of selected antioxidant compounds in *Thlaspi* species are shown in Table 2 and Figure 1. The structures of the ten most prevalent phenolic compounds in *Thlaspi* species are given in Figure 2.

LC–MS/MS profiling of the methanol extracts from nine *Thlaspi* species enabled the comprehensive identification and quantification of a wide range of phenolic and flavonoid compounds using 53 reference standards. The analysis revealed the presence of several biologically important phytochemicals, including quinic acid, protocatechuic acid, chlorogenic acid, cynaroside, rutin, isoquercitrin, astragalin, luteolin, apigenin, and kaempferol (Figure 2). Considerable variations in phenolic composition were observed among the investigated species, indicating species-dependent phytochemical diversity. Among the detected compounds, isoquercitrin, astragalin, cynaroside, and protocatechuic acid were identified as major constituents in several extracts.

### 2.3. Reducing Power

The ferric reducing antioxidant power assay is based on the reduction of Fe^3+^ to Fe^2+^ in the presence of antioxidant compounds contained in the *Thlaspi* extracts. Following the reduction process, the addition of ferric ions leads to the formation of the Perl’s Prussian Blue complex, Fe_4_[Fe(CN)_6_]_3_, which exhibits maximum absorbance at 700 nm [36,37]. An increase in absorbance indicates enhanced reducing power and, consequently, stronger antioxidant activity [38]. As summarized in Table 3 and Table 4 and Figure S1A, the Fe^3+^-reducing capacities of *Thlaspi* species extracts and standard antioxidants at 30 μg/mL were ranked in the following order: α-tocopherol (2.778 ± 0.248, r^2^ = 0.9999) > Trolox (2.334 ± 0.167, r^2^ = 0.9997) > BHA (2.319 ± 0.041, r^2^ = 0.9629) > BHT (1.873 ± 0.152, r^2^ = 0.9918) > *T. annuum* methanol extract (0.961 ± 0.011, r^2^ = 0.9795) > other *Thlaspi* methanol extracts > *T. annuum* water extract (0.480 ± 0.012, r^2^ = 0.9949) > *T. watsonii* methanol extract (0.354 ± 0.011, r^2^ = 0.9843) > other *Thlaspi* water extracts > *T. cariense* water extract (0.258 ± 0.022, r^2^ = 0.9985).

The Cu^2+^-reducing capacities of the *Thlaspi* species extracts were evaluated using the CUPRAC assay at a concentration of 30 μg/mL. Among the tested samples, the methanol extracts generally exhibited stronger reducing activity than the corresponding water ex-tracts. The highest Cu^2+^-reducing activity was observed for the methanol extract of *T. aghricum* (0.753 ± 0.023), whereas the lowest activity was detected in the water extract of *T. elegans* (0.241 ± 0.003) (Figure S1B). The results demonstrated that the Cu^2+^-reducing capacities of the extracts were positively associated with their phenolic and flavonoid contents.

The reducing capacities of *Thlaspi* species extracts and standard antioxidants were evaluated using the Fe^3+^–TPTZ-reducing power assay. According to the results presented in Table 4 and Figure S1C, the reducing activities of the tested samples at 30 μg/mL followed the decreasing order: α-Tocopherol (2.434 ± 0.103, r^2^ = 0.8714) > BHA (2.151 ± 0.020, r^2^ = 0.9367) > Trolox (2.108 ± 0.026, r^2^ = 0.9291) > BHT (2.031 ± 0.190, r^2^ = 0.9670) > *T. violascens* water extract (0.943 ± 0.022, r^2^ = 0.9881) > other *Thlaspi* water extracts > *T. annuum* methanol extract (1.007 ± 0.021, r^2^ = 0.9786) > other *Thlaspi* methanol extracts > *T. elegans* water extract (0.576 ± 0.010, r^2^ = 0.9585) > *T. arvense* methanol extract (0.566 ± 0.041, r^2^ = 0.9521).

The observed absorbance directly reflects the electron-donating ability of the tested extracts. The results demonstrated that both water and methanol extracts of *Thlaspi* species possess considerable reducing capacities, although their activities were lower than those of the standard antioxidants.

As shown in Table 2 and Table 3, significant positive correlations were observed between the phenolic and flavonoid contents of *Thlaspi* methanol and water extracts and their Fe^3+^ and Cu^2+^-reducing capacities. A moderate positive correlation was observed between total phenolic content and Fe^3+^-reducing activity (rs = 0.584 *, *p* = 0.011), whereas flavonoid content showed a strong positive correlation with Cu^2+^-reducing activity (rs = 0.723 **, *p* = 0.001). These findings indicate that increased polyphenolic content contributes substantially to the reducing power and overall antioxidant potential of *Thlaspi* species extracts.

Furthermore, the Mann–Whitney test revealed statistically significant differences between water and methanol extract groups with respect to total phenolic content, flavonoid content, Fe^3+^-reducing power, Cu^2+^-reducing capacity, and Fe^3+^–TPTZ-reducing activity. In general, methanol extracts containing higher levels of polyphenolic compounds exhibited stronger reducing and antioxidant activities compared with the corresponding water extracts.

### 2.4. Radical Scavenging and Metal Chelating

Both methanol and water extracts obtained from *Thlaspi* species demonstrated the ability to scavenge DPPH radicals, indicating their potential as natural antioxidant sources. The DPPH^•^ scavenging activities of the extracts were evaluated, and the corresponding IC_50_ values were determined as presented in Table 5 and Figure S2A. All *Thlaspi* extracts exhibited concentration-dependent radical scavenging activity. Among the tested extracts, the water extracts of *T. arvense* and *T. alliaceum* showed the strongest DPPH^•^ scavenging activity with an IC_50_ value of 173.3 μg/mL, while the *T. aghricum* methanol extract exhibited comparatively lower activity with an IC_50_ value of 231.0 μg/mL. In comparison, the standard antioxidants displayed substantially stronger radical scavenging capacities, with IC_50_ values of 15.8 μg/mL for BHA, 38.5 μg/mL for BHT, 14.4 μg/mL for α-tocopherol, and 11.7 μg/mL for Trolox. The obtained results indicate that although *Thlaspi* species extracts possess notable antioxidants and free radical scavenging properties, their activities were lower than the activities of the synthetic and standard antioxidant compounds used as references. Nevertheless, the observed DPPH^•^ scavenging activities suggest that the bioactive constituents present in *Thlaspi* extracts, particularly phenolic and flavonoid compounds, may contribute significantly to their antioxidant potential. The radical scavenging capacities of both methanol and water extracts are summarized in Table 5.

The ABTS^•+^ radical scavenging assay demonstrated that all *Thlaspi* extracts exhibited concentration-dependent antioxidant activity, although their effectiveness varied considerably among species and extraction solvents. The methanol extract of *T. annuum* showed the strongest ABTS^•+^ scavenging capacity with the lowest IC_50_ value (28.9 µg/mL), whereas the methanol extracts of *T. arvense* and *T. elegans* displayed comparably lower activities (IC_50_ = 77.0 µg/mL). Other extracts exhibited moderate to weak radical scavenging effects, with IC_50_ values generally ranging from 30.1 to 69.3 µg/mL (Figure S2B). Nevertheless, all *Thlaspi* extracts were less potent than the reference antioxidants, including Trolox, α-tocopherol, BHA, and BHT, which showed markedly lower IC_50_ values, confirming their superior ABTS^•+^ radical scavenging efficiency.

The DMPD^•+^ radical scavenging assay demonstrated variable antioxidant activities among the *Thlaspi* extracts. The methanol and water extracts of *T. annum* exhibited the strongest scavenging capacities (IC_50_ = 99.0 µg/mL), whereas the water extracts of *T. alliaceum* and *T. watsonii* showed same and weaker inhibition profiles (IC_50_ = 346.5 µg/mL) (Figure S2C). Nevertheless, all extracts displayed considerably lower DMPD^•+^ scavenging activity than the reference antioxidants, including Trolox, α-tocopherol, BHA, and BHT, which exhibited substantially lower IC_50_ values.

This color change indicates the ability of the bioactive compounds present in the *Thlaspi* extracts to donate electrons, thereby reducing Fe^3+^ to Fe^2+^ and demonstrating their ferric reducing antioxidant capacity. Based on the IC_50_ values, the radical scavenging and metal chelating activities of *Thlaspi* species extracts were evaluated in comparison with a standard antioxidant, including ascorbic acid and EDTA. The Bipyridyl–Fe^2+^ metal chelation assay demonstrated that the IC_50_ values of the extracts ranged from 15.4 to 63.0 μg/mL, indicating moderate to strong ferrous ion chelating capacities (Figure S2D). In comparison, the IC_50_ values of the reference compounds were determined as 20.4 μg/mL for ascorbic acid and 3.8 μg/mL for EDTA. Several *Thlaspi* extracts exhibited metal chelating activities comparable to, or in some cases stronger than, those of ascorbic acid. Extracts possessing lower IC_50_ values showed a greater affinity for Fe^2+^ ions, suggesting an enhanced ability to inhibit Fe^2+^-mediated Fenton reactions and thereby reduce oxidative damage caused by hydroxyl radical formation. Since transition metal ions play a critical role in the generation of reactive oxygen species, the observed chelating activity may contribute significantly to the overall antioxidant potential of the extracts. These findings demonstrate that *Thlaspi* species are promising natural sources of bioactive compounds with effective antioxidants and metal chelating properties. The results obtained are in good agreement with the data presented in Figure S2D and Table 5.

### 2.5. Enzyme Inhibition

The inhibitory effects of *Thlaspi* extracts against CA II isoenzyme varied markedly depending on both species and extraction solvent. As seen in Table 6, IC_50_ values ranged from 41.9 to 256.5 µg/mL, indicating considerable differences in inhibitory potency. Among the tested samples, the water extract of *T. cataonicum* exhibited the strongest CA II inhibition (IC_50_ = 41.9 µg/mL), followed by the methanol extract of *T. arvense* (IC_50_ = 52.0 µg/mL) and the water extract of *T. aghricum* (IC_50_ = 60.0 µg/mL). Several extracts showed weak or no detectable inhibitory activity, highlighting species- and solvent-dependent variability. Although all extracts were less potent than the reference inhibitor acetazolamide, the observed activities suggest that selected *Thlaspi* species may represent promising natural sources of CA II inhibitory compounds with potential antiglaucoma applications.

Inhibition of acetylcholinesterase (AChE) remains one of the most established therapeutic approaches for the symptomatic treatment of AD [39]. The AChE inhibitory activities of both methanol and water extracts obtained from nine *Thlaspi* species were evaluated to investigate their potential neuroprotective properties associated with AD management. All tested extracts exhibited concentration-dependent AChE inhibitory effects, with IC_50_ values ranging between 17.3 and 24.1 μg/mL (Table 6). Among the investigated samples, certain methanol extracts demonstrated comparatively stronger inhibition than that of the corresponding water extracts, suggesting that polyphenolic constituents extracted by methanol may contribute significantly to cholinesterase inhibition. The observed activities may be associated with the presence of flavonoids and phenolic compounds such as rutin, isoquercitrin, astragalin, and luteolin identified by LC–MS/MS analysis. These findings indicate that *Thlaspi* species may represent promising natural sources of bioactive compounds with potential anti-AD properties.

The α-amylase inhibitory activities of *Thlaspi* species extracts were evaluated, and the results are presented in Table 6. Both methanol and water extracts exhibited inhibitory effects against the α-amylase enzyme, with IC_50_ values ranging from 122.4 to 245.9 μg/mL. In general, water extracts demonstrated stronger inhibitory activity than that of the corresponding methanol extracts, indicating that highly polar phytochemicals may contribute significantly to α-amylase inhibition. Although the inhibitory activities of the extracts were lower than those of standard antidiabetic agents, the observed effects are nevertheless considerable and suggest noteworthy antidiabetic potential. Since α-amylase plays a crucial role in the hydrolysis of dietary starch into absorbable glucose molecules, inhibition of this enzyme may potentially delay carbohydrate digestion and reduce postprandial hyperglycemia. Therefore, the inhibitory activities observed in *Thlaspi* extracts may have potential relevance for the dietary management of T2DM.

To the best of our knowledge, this is the first comprehensive report describing the inhibitory activities of *Thlaspi* species extracts against α-amylase, AChE, and CA II. The observed enzyme inhibitory activities suggest that *Thlaspi* species constitute promising natural sources of bioactive phytochemicals with potential applications in functional foods, nutraceuticals, and complementary therapeutic strategies. These extracts may contribute to the management of postprandial hyperglycemia associated with T2DM, the symptomatic treatment of AD, and the reduction in intraocular pressure in glaucoma through CA II inhibition.

## 3. Discussion

The extraction yield is an important parameter reflecting the efficiency of the extraction process and the number of extractable constituents present in plant materials. In the present study, extraction yields varied considerably among the *Thlaspi* species investigated and between water and methanol extracts. Generally, water extracts produced higher yields than methanol extracts, indicating that a greater proportion of water-soluble constituents was present in the aerial parts of these species. The differences observed in extract amount and yield may be attributed to species-specific phytochemical composition, polarity of the extraction solvent, and the relative abundance of soluble metabolites. These findings demonstrate the influence of solvent selection on extraction efficiency and phytochemical recovery.

The total phenolic and flavonoid contents of the water and methanol extracts of *Thlaspi* species were determined to range from 8.29 to 49.14 mg GAE/g extract and from 2.33 to 74.66 mg QE/g extract, respectively. Previous studies have also reported considerable phenolic and flavonoid content in *Thlaspi* species. In a study investigating Chinese medicinal herbs, the total phenolic and flavonoid contents of the stem and leaf extracts of *Thlaspi arvense* were reported as 41.58 ± 1.68 mg GAE/g DW and 2.95 ± 0.38 mg CE/g DW, respectively [36]. Similarly, aqueous and methanol extracts of *T. arvense* collected from Bhutan were evaluated for the first time with respect to their phenolic composition and antioxidant potential. In that study, the aqueous extract contained 5.17 ± 0.25 μg GAE/mg extract of total phenolics and 17.00 ± 0.23 μg QE/mg extract of total flavonoids, whereas the methanol extract contained 32.64 ± 0.43 μg GAE/mg extract of total phenolics and 59.39 ± 2.99 μg QE/mg extract of total flavonoids [37]. Consistent with these previous reports, the present findings corroborate the finding that *Thlaspi* species possess substantial amounts of phenolic and flavonoid constituents. The detailed total phenolic and flavonoid contents of the *Thlaspi* extracts are presented in Table 1. Also, these findings correlate with the observed antioxidant and enzyme inhibitory activities of the *Thlaspi* species extracts.

The phytochemical findings were further correlated with antioxidant capacities determined by multiple complementary methods, including radical scavenging assays (DPPH^•^ and ABTS^•+^) and reducing power assays such as FRAP, Fe^3+^-reducing, and Cu^2+^-reducing tests, which are widely recommended for comprehensive antioxidant assessment [40,41,42]. In addition, the enzyme inhibitory potentials of the extracts were evaluated against α-amylase, AChE, and CA II, which are key enzymes associated with diabetes mellitus, AD, and glaucoma, respectively. These enzymes are known targets of numerous plant-derived phenolic compounds [43]. IC_50_ values were calculated using nonlinear regression analysis to ensure accurate determination of inhibitory potency. Overall, this study integrates phytochemical profiling with functional bioactivity analyses, allowing a comparative evaluation of solvent-dependent extraction efficiency and the relationship between phenolic composition and biological activity. Since solvent polarity significantly affects phytochemical extraction yield and biological properties, comparing methanol and water extracts provides valuable insight into the distribution of bioactive constituents within *Thlaspi* species [44,45]. Collectively, the findings demonstrate that *Thlaspi* species may serve as promising natural sources of multifunctional bioactive compounds with antioxidant and enzyme inhibitory properties.

Flavonoids, one of the major classes of phenolic compounds, are widely distributed in plants and possess diverse biological activities, including antioxidants, antimicrobial, anti-inflammatory, and enzyme inhibitory properties. Their remarkable antioxidant effects are mainly attributed to their ability to scavenge free radicals and inhibit the formation of reactive oxygen species [46,47,48]. In the present study, both methanol and water extracts of *Thlaspi* species were found to contain considerable amounts of total phenolics and flavonoids. These phytochemical constituents appear to contribute directly to the antioxidant capacities of the extracts. Furthermore, the results demonstrated that both solvent systems yielded extracts with substantial polyphenolic content, although methanol extracts generally exhibited higher levels of phenolic compounds and correspondingly stronger biological activities. When the total phenolic and total flavonoid contents were evaluated, both the phenolic and flavonoid contents in each methanol extract were found to be statistically significantly higher than those in the aqueous extracts at the *p* < 0.001 level.

The reduction capacity of a molecule might be a significant indicator of its possible antioxidant action. Antioxidant substances can donate electrons to reactive radicals, converting them into more stable and unreactive structures [49,50]. The potential of *Thlaspi* species phenolic compounds was determined with Fe^3+^ ions, Cu^2+^ ions, and Fe^3+^–TPTZ ions, in terms of reducing capabilities. The radical scavenging activity of *Thlaspi* species extracts was tested by DPPH and ABTS methods. Natural substances from *Thlaspi* species may have reducing qualities, which would neutralize ROS and oxidants.

The reducing abilities of the extracts were determined using a modified ferric reducing method originally described by Oyaizu [51], which remains a standard approach for assessing antioxidant potential in plant-derived samples. Increased absorbance values directly correlate with reducing capacity, reflecting the presence of potent reductants such as phenolics and flavonoids. This method has been applied in recent studies to evaluate plant antioxidant systems and correlate redox activity with phytochemical composition [52,53]. In the present study, both methanol and water extracts of *Thlaspi* species demonstrated concentration-dependent Cu^2+^-reducing activity, indicating the presence of electron-donating bioactive compounds, particularly phenolics and flavonoids [54,55].

Antioxidant capability is frequently evaluated using the DPPH∙ test, which is based on converting DPPH∙ to the non-radical form DPPH-H [56]. However, in comparison with the results of the different studies given, their effects were shown to be valuable. *T. arvense*’s stem and leaf extract scavenged DPPH radicals in 80.08 ± 0.46% in a different study, using a procedure where 200 μL (10 mg/mL, 15% aqueous ethanol) of the diluted extract was added to 3.80 mL of methanolic DPPH solution [36]. When DPPH radical scavenging activity (%) was tested at five distinct quantities (5–25 μg/mL), it was found to be 41.92 ± 0.31 for aqueous extract and 47.23 ± 0.24 for methanol extract of *T. arvense* species, which was studied before as a high-altitude medicinal plant [37]. In another study, the capacity of pennycress seed oils to scavenge free radicals was compared and reported. The *T. arvense* seed oil showed EC_50_ values of 8.65–19.21 mg/mL for DPPH radical-scavenging activity and 6.82–10.61 mg/mL for ABTS^•+^ scavenging activity across five distinct geographic regions [16]. In this study, it was observed that five species (*T. alliaceum*, *T. arvense*, *T. violascens*, *T. aghricum*, and *T. watsonii*) exhibited scavenging activity in all antioxidant assays (DPPH, ABTS, DMPD, and metal chelation) when either aqueous or methanol extracts were used. They are qualitatively comparable. The other species did not exhibit activity in at least one of these methods. The inhibition values of the *Thlaspi* species methanol and water extracts were lower, but comparable to the reference value, compared to tacrine, a common reference inhibitor of the AChE.

Unlike the other *Thlaspi* species studied, *T. arvense*, *T. aghricum*, *T. cataonicum*, and *T. annuum* also inhibited the CAII enzyme. A limitation of our study is that we collected only a single sample from each species. More sensitive measurements could improve the reliability of the results. To the best of our knowledge, this study is the first to report on the inhibitory effects of *Thlaspi* species extracts against the CA II isoenzyme. These findings suggest that *Thlaspi* species possess promising bioactive constituents with potential therapeutic relevance. Although the present results are limited to in vitro investigations, the observed antioxidant and enzyme inhibitory activities indicate that *Thlaspi* extracts may serve as potential natural candidates for further studies targeting diabetes mellitus, AD, and glaucoma. Therefore, comprehensive vivo, mechanistic, and pharmacological studies are warranted to elucidate their therapeutic potential and possible clinical applications.

## 4. Materials and Methods

### 4.1. Chemicals

Acetylcholinesterase, acetylcholine iodide, α-glycosidase, p-nitrophenyl-D-glucopyranoside, 1,1-diphenyl-2-picryl-hydrazyl (DPPH), 2,2-azino-bis 3-ethylbenzthiazoline-6-sulfonic acid (ABTS), neocuproine (2,9-dimethyl-1,10-phenanthroline), butylated hydroxytoluene (BHT), N,N-dimethyl-p-phenylenediamine (DMPD), butylated hydroxyanisole (BHA), trichloroacetic acid (TCA), α-tocopherol, Trolox, nitroblue tetrazolium (NBT), 3-(2-pyridyl)-5,6-bis(4-phenyl-sulfonic acid)-1,2,4-triazine (Ferrozine) and standard phenolic compounds of LC-MS/MS were purchased from Sigma (Sigma-Aldrich GmbH, Sternheim, Germany). Ammonium thiocyanate was purchased from Merck (Darmstadt, Germany). Due to methanol having inhibitory effects on some enzymes, *Thlaspi* species methanol and water extracts were dissolved in methanol for the antioxidant activities, but in DMSO for the enzyme inhibition experiments.

### 4.2. Plant Materials

Dr. Mehmet Cengiz Karaismailoğlu gathered and identified plant species from various phytogeographic areas of Turkey throughout the flowering time. In addition to Karaismailoğlu’s collection, specimens are kept in the Siirt University Flora and Fauna Center (SUFAF). Table 7 shows the species of *Thlaspi* that were analyzed along with their localities.

### 4.3. Preparation of the Methanol Extract of Thlaspi Species

The extraction procedure of ethanol and water extracts of *Thlaspi* species was performed as previously described [57]. Water extracts of the specimens were performed using 400 mL of distilled water, and 40 g of dehydrated *Thlaspi* species water extract was ground in a mill (Table 7). This mixture was boiled for thirty minutes using a magnetic stirrer. Then, to remove water, the extracts were lyophilized in a Lyophilizator at a temperature of −80 °C under a pressure of 5 mm-Hg (Labconco, Freezone). This process took 6 days. For the methanol extracts of the specimens, 40 g of dried *Thlaspi* species was pulverized before being combined with 400 mL methanol and stirred on a magnetic stirrer for an hour. It was filtered first using ordinary filter paper and then using blue-band filter paper. After the extracts were filtered, the filtrates were collected. The excessive amount of methanol in the filtrate was removed using a rotary evaporator (RE 100 Bibby, Stone Staffordshire, UK) running at 50 °C. All extracts were stored before being studied in experimental research, at −20 °C in a dark plastic container.

### 4.4. Determination of the Total Phenolic Contents

Analysis of the phenolic content in methanol and water extracts of *Thlaspi* species was performed using the Folin–Ciocalteu reagent according to a putative methodology [58], with slight modifications to the approach [59]. We poured the appropriate 0.5 mL of each extracted sample into the 1.0 mL Folin–Ciocalteu reagent. The solution was then thoroughly mixed and neutralized with carbonate (0.5 mL, 1%). The absorbance at 760 nm was recorded towards a blank specimen made up of distilled water after 2 h of incubation in the dark at room temperature. The linear regression equation of the developed gallic acid calibration curve was used to determine the phenolic contents. The phenolic content analysis results were expressed in milligrams of gallic acid equivalents (GAE) for each gram of extract from *Thlaspi* species.

### 4.5. Determination of the Total Flavonoid Contents

Flavonoids, also extensively found in plants, are the most prevalent polyphenolic chemicals in the typical human diet. An assay for colorimetry based on the formerly reported process [60] was used to measure total flavonoids in methanol and water extracts of *Thlaspi* species. A mixture of 1.5 mL of 95% methanol and 0.5 mL of the methanol and water extracts of *Thlaspi* species were prepared. Then, 1.5 mL of Al(NO_3_)_3_ (10%), 0.5 mL of CH_3_COOK (1.0 M), and 2.3 mL of distilled water were added to the specimens, and they were then stirred vigorously. The specimens that were stirred were then maintained in a dark at room temperature for forty minutes. The absorbance was measured at 415 nm. Distilled water was used as a blank and control. A calibration curve for quercetin was created, and the linear regression equation of the calibration curve was used to estimate flavonoid content. Quercetin equivalents (QE), which are the results, are expressed as mg per gram of *Thlaspi* species extract. Table 7 indicates the phenolic and flavonoid contents of methanol and water extracts of *Thlaspi* species.

### 4.6. LC-MS/MS Analysis

In a volumetric flask, 100 mg of methanol and water extracts of *Thlaspi* species were dissolved in 5 mL of methanol–water (50:50 *v*/*v*). One milliliter of the mixture was then transferred into a second volumetric flask, which held 5 mL. Then, 100 μL of *Thlaspi* species methanol and water extracts were added and diluted to the volume with methanol–water (50:50 *v*/*v*). A vial with a cap was filled with a 1.5 mL aliquot of the final solution, and 10 μL of the sample was put into the LC-MS/MS. The autosampler’s samples were maintained at 15 °C throughout the testing [61].

The LC-MS/MS analysis used in this study was carried out according to the research conducted by Yılmaz [62] at the Central Research Laboratory of Dicle University. The method was adapted to the *Thlaspi* species. Phytochemical standards were purchased from Sigma-Aldrich (Steinheim, Germany) and used to investigate the phytochemical components in *Thlaspi* species water and methanol extracts.

### 4.7. Reducing Ability Assays

The Fe^3+^-reducing ability of methanol and water extracts of *Thlaspi* species is determined by the Oyaizu method [51]. Briefly, various concentrations of methanol and water extracts of *Thlaspi* species (10–30 μg/mL) are added to the same volume of phosphate buffer (1.25 mL, pH: 6.6; 0.2 M) and K_3_Fe(CN)_6_ solution (1%). After 30 min of incubation at 50 °C, the mixture is acidified with 1.25 mL trichloroacetic acid (10%). Before determining the absorbances of the methanol and water extracts of *Thlaspi* species at 700 nm, an aliquot of 0.5 mL FeCl_3_ (0.1%) solution is poured. Phosphate buffer was used as a blank.

With minor modifications provided in detail, the Cu^2+^-reducing effects of methanol and water extracts of *Thlaspi* species were measured [63], in an approach primarily adapted from that of Apak et al. [64]. To accomplish this, the same volumes of 250 µL of the methanol and water extracts of *Thlaspi* species solutions (10–30 µg/mL) in a test tube were joined to the same amount of the neocuproine mixture (7.5 mM), acetate buffer (0.25 mL, 1.0 M), and CuCl_2_ solution (10 mM). The total volume of the liquid was adjusted to 2 mL using distilled water and vigorous shaking. The glass tubes were then sealed and maintained at 25 °C until utilized in research. After 30 min, they were spectrophotometrically measured at 450 nm. An acetate buffer mixture was utilized as a blank specimen. A higher absorbance of the reaction mixture indicates a greater capability for Cu^2+^ reduction.

The FRAP-reducing power is based on the Fe^3+^–TPTZ reduction in acidic solution. At 593 nm, the reduced form of Fe^2+^-TPTZ is detected spectrophotometrically [65]. The FRAP reagent mixture comprised TPTZ solution (10 mM, 2.25 mL) and FeCl_3_ (20 mM, 2.25 mL) in buffer (2.5 mL, pH 3.6, 0.3 M). The absorbance was recorded at 593 nm after a 0.2 mL portion of the sample was added to 1.8 mL of FRAP reagent. A blank specimen was accomplished using a phosphate-buffered solution.

### 4.8. Radical Scavenging Activities

#### 4.8.1. DPPH Radical Scavenging Activity

Decolorization of the purple DPPH mixture in methanol reveals the existence of pure chemical substances with hydrogen atom or electron-donating characteristics. The solution used for this spectrophotometric experiment is the stable DPPH radicals [65,66]. DPPH^•^ scavenging activity of *Thlaspi* species extracts was realized according to the Blois technique [67]. This method was used with minor changes to evaluate the DPPH^•^ radical scavenging capability of methanol and water extracts of *Thlaspi* species, while a stable free radical known as DPPH was tested for discoloration at a particular wavelength. The DPPH solution was prepared the day before the measurement [68]. The beaker containing the mixture was covered with aluminum foil and swirled at 4 °C for 16 h in the dark. Shortly after making a 10 mM DPPH solution in methanol, 0.5 mL was mixed with 2 mL of *Thlaspi* species extracts solution in methanol at various concentrations (10–30 µg/mL). The *Thlaspi* species samples were swirled and incubated in the dark at 30 °C for 30 min. The absorbance at 517 nm was measured with the blank samples. A decrease in absorbance indicates the activation of DPPH free radicals. The decreased absorbance demonstrates that DPPH radicals were removed by antioxidant compounds in methanol and water extracts of *Thlaspi* species.

#### 4.8.2. ABTS^•+^ Scavenging Activity

ABTS, a stable free radical, is lightning even in its non-radical form. The spectrophotometric approach of Re et al. [69] was used to determine the analysis of ABTS^•+^ scavenging capability of methanol and water extracts of *Thlaspi* species. This method includes introducing an antioxidant to a prepared ABTS radical solution, and the remaining ABTS^•+^ was recorded spectrophotometrically at 734 nm after a predetermined length of time. To make ABTS^•+^, 2.45 mM potassium persulfate (K_2_S_2_O_8_) and 2 mM ABTS in water were mixed. The mixture was then incubated for 6 h at room temperature and in the dark. Although the absorbance took more than 6 h to peak and stabilize, the ABTS began to oxidize immediately. The radical cation remains stable in this form for more than two days when kept at room temperature and in the dark. To conduct the test, the solution was diluted in phosphate buffer (pH 7.4) to procure an absorbance of 0.700 ± 0.02 at 734 nm in a 1 mL cuvette. Then, this was allowed to equilibrate at 30 °C, which is the standard temperature for conducting experiments. Next, 1 mL of the ABTS^•+^ mixture was mixed with 3 mL of *Thlaspi* species solutions in methanol at various concentrations (10–30 µg/mL). After 30 min of mixing the sample, the absorbance was determined, and the rate of radical scavenging was recorded for each concentration compared to a blank devoid of scavenger [70]. The degree of lightning was ascertained by calculating the ratio of decrease in absorbance.

#### 4.8.3. DMPD^•+^ Scavenging Activity

The radical scavenging ability of methanol and water extracts of *Thlaspi* species towards DMPD was assessed using the methodology of Fogliano et al. [71]. The extract’s capacity to reduce the generation of DMPD^•+^ cation radicals is the basis for this assessment. Briefly, 5 mL of distilled water was utilized to dissolve 105 mg of DMPD. After that, 1 mL of this mixture was added to 100 mL of pH 5.3, 0.1 M acetate buffer, and was mixed for 5 min in the dark. By adding 0.2 mL ferric chloride (0.05 M) to this composition, DMPD^•+^ was formed. The solution was adjusted with 0.5 mL of distilled water after standard antioxidants, methanol, and water extracts of *Thlaspi* species were added at different quantities (10–30 µg/mL). The reaction combination received an immediate addition of one milliliter of DMPD^•+^ solution. After a tentative combination of the reaction solution, the mixtures were incubated in a dark room for 50 min and the absorbance was measured at 505 nm [72].

#### 4.8.4. Metal Chelating Assay

The metal chelating assay by 2,2′-bipyridine was realized according to the methods of Re et al. [69]. Various quantities of methanol and water extracts of *Thlaspi* species were added to a 0.25 mL FeSO_4_ solution (2 mM). As a result, the sample and Fe^2+^ ions were allowed to interact. Consequently, the *Thlaspi* species sample chelates Fe^2+^ ions. Subsequently, the mixture was blended with 1.5 mL of 2,2′-bipyridine (0.2%) solution dispersed in 1 mL of Tris-HCl solution (pH 7.4) and 0.2 M of HCl. Then, 2.5 mL of ethyl alcohol and 0.63 mL of deionized water were added to the solution after it had been incubated for 30 min. Their absorbances were calculated at 522 nm in comparison to a blank made of Tris-HCl buffer.

### 4.9. Enzyme Inhibition Studies

#### 4.9.1. AChE Inhibition Study

The cholinergic enzyme inhibitory activities of *Thlaspi* species extracts were determined using Ellman’s method [73]. The AChE serum derived from electric eels was used to accomplish this. Simply put, a concentration of 10–30 µg/mL of an extract from a certain *Thlaspi* species is conveyed to an enzyme mixture (50 μL, 5.32 × 10^−3^ EU) in a buffer (1.0 M Tris/HCl, 100 μL, pH 8.0). For ten minutes, the combinations were kept at 20 °C. Following that, 50 μL of a mixture comprising acetylthiocholine iodide (AChI) and DTNB (5,5′-dithio-bis(2-nitro-benzoic acid)) (0.5 mM) was assessed. After starting the reaction, the mixture’s absorbance at 412 nm was recorded using spectrophotometry [74].

#### 4.9.2. α-Amylase Inhibition Study

The inhibitory effects of methanol and water extracts of *Thlaspi* species on α-amylase were measured according to Xiao et al. [75]. In brief, 1 g of starch was dissolved in 40 mL of 0.4 M alkaline solution and heated to 80 °C for 30 min. Once the mixture cooled, its pH was equilibrated to 6.9, and its total volume was brought to 100 mL using deionized water. Next, a mixture of 35 μL of starch and phosphate buffer (pH 6.9) was mixed with various amounts of methanol and water extracts of *Thlaspi* species. Afterward, we transferred 20 μL of the α-amylase solution to the finished combination and incubated it for 1 h at 35 °C. The reaction was finally terminated when 50 μL of 0.1 M HCl was added. Sample absorbance was then recorded at 580 nm using a blank sample with phosphate-buffered solution.

#### 4.9.3. CA II Isoenzyme Inhibition Study

As mentioned, CA II isoenzymes were isolated and purified by employing human erythrocytes discarded after laboratory analysis and Sepharose-4B-L-Thyrosine sulfanilamide affinity chromatography [76]. Throughout the enzyme purification approach, protein concentrations were measured using Bradford’s technique at 595 nm [77]. To perform esterase activity, the method of Verpoorte et al. was used [78]. The absorbance was measured at 348 nm using a spectrophotometer (Shimadzu, UVmini-1240 UV–VIS, Tokyo, Japan). As a reference standard, acetazolamide (AZA) was utilized.

#### 4.9.4. Determination of IC_50_ Value

The inhibitory potency of methanol and water extracts of *Thlaspi* species was measured using IC_50_ values. The graphs produced by the enzyme activity corresponding to rising concentrations of methanol and water extracts of *Thlaspi* species were utilized to compute the IC_50_ values [79].

#### 4.9.5. Statistical Analysis

The results of each analysis were repeated three times for each experiment. The results are given as mean ± SD. An independent samples t-test was performed for evaluation of two extract groups; significant variations were regarded as *p* < 0.05. The coefficient of determination (r^2^) was calculated from the linear regression between the experimentally measured values and the corresponding model-predicted values using the least-squares regression method. The r^2^ value represents the proportion of the variance in the experimental data explained by the regression model, where a value closer to 1 indicates a stronger agreement between the predicted and experimental results.

## 5. Conclusions

*Thlaspi* species have bioactive content of various qualities and amounts of secondary metabolites (phenolics and flavonoids); *Thlaspi* plants are known and used traditionally, although they have not been studied. In this study, it was first indicated that the water and methanol extracts of endemic *Thlaspi* plant species were shown to have antioxidant activities and had inhibitory effects on AChE, α-amylase, and CA II enzymes. *Thlaspi* plants represent valuable botanical sources with the phenolic and flavonoids they contain, which can be used as a natural product in the treatment of serious and common T2DM, AD, glaucoma, and similar diseases, which are open to research in the food and pharmaceutical industries. For years, humans have customarily ingested *Thlaspi* species in the wild. The usage of synthetic antioxidants has been limited in recent years; it has been investigated in the context of alternative research. The phenolic and flavonoid chemicals found in *Thlaspi* species extracts can be liberated and employed in medicine by application of various extraction techniques, as demonstrated by in vitro, animal trials, cell cultures, interventional studies, and future perspectives. Both alone and in combination, these isolated chemicals have potential uses. The phenolic and flavonoid secondary metabolite contents in the aerial parts are mild and adequate; compounds found in plant extracts are also important in enzyme inhibition studies. We recognized that *Thlaspi* species, which have several endemic plants, have antioxidants, antidiabetic, anti-AD, and anti-glaucoma effects. Based on the LC-MS/MS analysis, the primary compounds found in *Thlaspi* species methanol extracts were quinic acid, gallic acid, protocatechuic acid, chlorogenic acid, vanillin, piceid, cynaroside, rutin, isoquercitrin, hesperidin, cosmosiin, astragalin, Nicotiflorin, and luteolin. Furthermore, the *Thlaspi* species methanol and water extracts had comparable effective antioxidant activity, reducing power, phenolic contents, and inhibition compared to AChE, α-glycosidase, and hCA II in comparison to inhibitors of these enzymes. Except for one or two non-endemic species, almost all the endemic species studied in detail in this study have been studied and are reported in the literature for the first time. Enzyme inhibitors can have important effects in the prevention of common diseases when their use is adjusted as a food additive, like the *Thlaspi* species used as biodiesel and food. Clinical pharmacology investigations can support the pharmacological use of *Thlaspi* species extracts in patients with disorders.

Despite the promising findings, several limitations of the present study should be acknowledged. All biological activities were evaluated exclusively using in vitro biochemical assays; therefore, the observed effects cannot be directly extrapolated to in vivo therapeutic efficacy. Moreover, the bioavailability, pharmacokinetic behavior, metabolism, and safety of the *Thlaspi* extracts and their bioactive constituents were not investigated. Although LC–MS/MS analysis provided comprehensive phytochemical profiling, the specific compounds responsible for the observed antioxidant and enzyme inhibitory activities were not identified through bioactivity-guided fractionation or isolation. Furthermore, the molecular mechanisms underlying the inhibition of α-amylase, acetylcholinesterase, and carbonic anhydrase II were not explored. Therefore, further in vivo pharmacological studies, toxicological evaluations, bioactivity-guided isolation of active constituents, and mechanistic investigations are required to validate the therapeutic potential of *Thlaspi* species and support their development as functional food ingredients or nutraceuticals.

## Figures and Tables

**Table 1:** Extraction conditions, extraction yields, total phenolic and total flavonoid contents of the examined *Thlaspi* species.

No	Taxa	Water Extract				Methanol Extract			
		Amount (g)	Yield (%)	Total Phenolics ^§^	Total Flavonoids ^ψ^	Amount (g)	Yield (%)	Total Phenolics ^§^	Total Flavonoids ^ψ^
1	*Thlaspi alliaceum*	2.32	5.8	13.43	26.00	0.81	2.0	34.86	74.66
2	*Thlaspi arvense*	6.22	15.6	8.29	21.00	2.10	5.3	21.43	43.00
3	*Thlaspi violascens* *	4.36	10.9	24.29	17.33	2.20	5.5	40.00	34.33
4	*Thlaspi aghricum* *	2.75	6.9	12.00	22.33	0.20	0.5	32.29	51.66
5	*Thlaspi cataonicum*	3.90	9.8	11.43	28.67	2.56	6.4	34.29	68.00
6	*Thlaspi annuum*	4.53	11.3	41.71	30.67	0.53	1.3	28.86	40.33
7	*Thlaspi watsonii* *	2.80	7.0	10.86	29.33	1.60	4.0	49.14	48.33
9	*Thlaspi cariense* *	5.27	13.2	18.29	2.33	2.10	5.3	16.29	64.33
12	*Thlaspi elegans* *	5.38	13.5	18.57	25.00	1.40	3.5	14.29	31.67

*: Endemic taxon, **^§^** They were expressed as µg GAE/mg extract, **^ψ^** They were expressed as µg QE/mg extract. The levels of phenolics and flavonoids were found to be statistically significantly higher (*p* < 0.001) in the methanol extract than in the aqueous extract.

**Table 2:** LC–MS/MS parameters of selected antioxidant compounds in 1: *T. alliaceum*, 2: *T. arvense*, 3: *T. violascens*, 4: *T. aghricum*, 5: *T. cataonicum*, 6: *T. annuum*, 7: *T. watsonii*, 9: *T. cariense* and 12: *T. elegans* chromatograms of methanol extracts in mg/g analyte concentration (N.D.: Not detected).

No	Analytes	1	2	3	4	5	6	7	9	12
1	Quinic acid	2.039	1.696	0.406	2.247	1.524	1.802	2.161	8.551	5.974
2	Fumaric acid	0.315	N.D.	N.D.	N.D.	N.D.	N.D.	N.D.	0.298	N.D.
3	Aconitic acid	0.02	0.018	0.003	N.D.	0.011	0.017	N.D.	0.011	N.D.
4	Gallic acid	0.059	0.033	1.05	5.345	0.025	0.5	N.D.	0.08	0.312
6	Protocatechuic acid	0.185	0.472	9.77	51.678	0.115	2.454	0.082	1.139	8.283
8	Gentisic acid	0.043	0.398	N.D.	N.D.	0.178	0.124	0.086	0.340	0.559
9	Chlorogenic acid	0.037	0.082	0.529	0.158	0.252	7.225	0.905	0.019	0.040
10	Protocatechuic aldehyde	N.D.	N.D.	0.029	0.059	N.D.	0.071	N.D.	0.04	0.012
11	Tannic acid	0.148	0.027	0.026	0.059	N.D.	0.131	N.D.	0.014	N.D.
14	*p*-OH benzoic acid	N.D.	0.171	N.D.	0.669	N.D.	N.D.	N.D.	0.289	0.786
17	Caffeic acid	N.D.	0.145	0.029	0.074	N.D.	N.D.	N.D.	N.D.	N.D.
19	Vanillin	N.D.	N.D.	N.D.	2.078	N.D.	N.D.	N.D.	N.D.	N.D.
20	Syringic aldehyde	N.D.	N.D.	N.D.	0.115	N.D.	N.D.	N.D.	N.D.	N.D.
23	Piceid	N.D.	N.D.	N.D.	1.071	0.079	N.D.	N.D.	N.D.	N.D.
24	*p*-Coumaric acid	0.011	0.314	0.041	0.201	N.D.	0.249	N.D.	0.135	0.247
28	Coumarin	N.D.	N.D.	N.D.	0.017	N.D.	N.D.	N.D.	N.D.	N.D.
29	Salicylic acid	0.029	0.104	0.704	0.079	0.032	0.02	0.065	0.047	0.102
30	Cynaroside	0.711	14.638	0.371	3.553	1.195	2.022	57.456	0.548	0.146
31	Miquelianin	N.D.	N.D.	N.D.	N.D.	N.D.	0.023	N.D.	N.D.	N.D.
33	Rutin	N.D.	N.D.	0.360	0.059	N.D.	1.366	2.621	10.004	0.026
34	Isoquercitrin	0.541	1.729	7.397	15.549	0.888	8.063	0.536	63.006	4.010
35	Hesperidin	0.004	0.009	0.003	0.028	0.015	0.642	0.730	5.574	0.013
40	Cosmosiin	0.134	3.931	0.017	0.505	0.341	0.264	16.434	0.389	0.005
42	Astragalin	0.069	1.905	0.214	5.892	0.013	3.069	1.969	74.159	0.324
43	Nicotiflorin	N.D.	N.D.	0.019	0.085	N.D.	0.043	0.855	14.534	N.D.
47	Quercetin	N.D.	N.D.	0.055	0.092	N.D.	N.D.	N.D.	0.436	0.198
48	Naringenin	N.D.	0.021	N.D.	0.162	N.D.	N.D.	N.D.	N.D.	0.005
50	Luteolin	0.006	1.315	0.007	0.143	0.005	0.007	0.295	0.018	0.015
51	Genistein	N.D.	N.D.	N.D.	N.D.	N.D.	N.D.	N.D.	0.014	N.D.
52	Kaempferol	N.D.	0.029	0.009	0.026	N.D.	N.D.	N.D.	0.533	0.498
53	Apigenin	0.003	0.399	0.003	0.041	0.006	0.001	0.04	0.014	0.003
54	Amentoflavone	N.D.	N.D.	N.D.	N.D.	N.D.	N.D.	0.018	N.D.	N.D.
55	Chrysin	N.D.	0.002	N.D.	0.003	N.D.	N.D.	0.008	N.D.	N.D.
56	Acacetin	N.D.	N.D.	N.D.	0.004	N.D.	N.D.	0.026	0.310	N.D.

**Table 3:** Given test parameters, Spearman’s correlation analysis.

Spearman’s rho		Total Phenolic Compounds (mg/mL)	Total Flavonoid Compounds (mg/mL)	Fe^3+^ Reducing ^§^	Cu^2+^ Reducing ^§^	Fe^3+^_–_TPTZ Reducing ^§^
Total phenolic content	Correlation coefficient	1.000	0.558 *	0.584 *	0.509 *	0.437 0.070
	Sig. (2-tailed)	-	0.016	0.011	0.031	
Total flavonoid content	Correlation coefficient	0.558 *	1.000	0.571 *	0.723 **	0.397
	Sig. (2-tailed)	0.016	-	0.013	0.001	0.103
Fe^3+^-reducing ^§^	Correlation coefficient	0.584 *	0.571 *	1.000	0.755 **	0.617 **
	Sig. (2-tailed)	0.011	0.013	-	0.000	0.006
Cu^2+^-reducing ^§^	Correlation coefficient	0.509 *	0.723 **	0.755 **	1.000	0.635 **
	Sig. (2-tailed)	0.031	0.001	0.000	-	0.005
Fe^3+^–TPTZ-reducing ^§^	Correlation coefficient	0.437	0.397	0.617 **	0.635 **	1.000
	Sig. (2-tailed)	0.070	0.103	0.006	0.005	-

^§^: They were expressed as absorbance, *: Correlation is significant at the 0.05 level (2-tailed), **: Correlation is significant at the 0.01 level (2-tailed).

**Table 4:** Cupric ion (Cu^2+^)-reducing, ferric ion (Fe^3+^)-reducing and FRAP reduction results of water and ethanol extracts of nine *Thlaspi* species at 30 µg/mL concentration. The results are given as mean ± SD.

Antioxidants	Fe^3+^-Reducing				Cu^2+^-Reducing				Fe^3+^–TPTZ-Reducing			
	Water Extract		Methanol Extract		Water Extract		Methanol Extract		Water Extract		Methanol Extract	
	λ_700_	r^2^	λ_700_	r^2^	λ_450_	r^2^	λ_450_	r^2^	λ_593_	r^2^	λ_593_	r^2^
BHA	2.319 ± 0.041	0.9629	2.319 ± 0.041	0.9629	2.849 ± 0.020	0.9994	2.849 ± 0.020	0.9994	2.151 ± 0.020	0.9367	2.151 ± 0.020	0.9367
BHT	1.873 ± 0.152	0.9918	1.873 ± 0.152	0.9918	2.865 ± 0.038	0.9991	2.865 ± 0.038	0.9991	2.031 ± 0.190	0.967	2.031 ± 0.190	0.967
Trolox	2.334 ± 0.167	0.9997	2.334 ± 0.167	0.9997	2.555 ± 0.022	0.9987	2.555 ± 0.022	0.9987	2.108 ± 0.026	0.9291	2.108 ± 0.026	0.9291
α-Tocopherol	2.778 ± 0.248	0.9999	2.778 ± 0.248	0.9999	2.185 ± 0.110	0.9986	2.185 ± 0.110	0.9986	2.434 ± 0.103	0.8714	2.434 ± 0.103	0.8714
*T. alliaceum*	0.319 ± 0.008	0.9999	0.421 ± 0.009	0.9938	0.261 ± 0.007	0.9999	0.562 ± 0.018	0.9989	0.679 ± 0.017	0.9772	0.890 ± 0.003	0.9912
*T. arvense*	0.327 ± 0.022	0.9888	0.548 ± 0.027	0.9926	0.271 ± 0.039	0.9903	0.399 ± 0.014	0.9957	0.652 ± 0.019	0.9645	0.566 ± 0.041	0.9521
*T. violascens*	0.448 ± 0.010	0.9911	0.761 ± 0.054	0.9926	0.356 ± 0.005	0.9927	0.608 ± 0.040	0.9965	0.943 ± 0.022	0.9881	0.867 ± 0.025	0.9843
*T. aghricum*	0.402 ± 0.014	0.9999	0.831 ± 0.017	0.9946	0.311 ± 0.016	0.9854	0.753 ± 0.023	0.9936	0.838 ± 0.012	0.9566	0.834 ± 0.021	0.9935
*T. cataonicum*	0.350 ± 0.000	0.9923	0.693 ± 0.033	0.9953	0.340 ± 0.009	0.9960	0.497 ± 0.012	0.9774	0.625 ± 0.021	0.9566	0.962 ± 0.037	0.9823
*T. annuum*	0.480 ± 0.012	0.9949	0.961 ± 0.011	0.9795	0.503 ± 0.016	0.9999	0.504 ± 0.071	0.9939	0.724 ± 0.012	0.9843	1.007 ± 0.021	0.9786
*T. watsonii*	0.332 ± 0.004	0.9906	0.354 ± 0.011	0.9843	0.466 ± 0.034	0.9855	0.328 ± 0.006	0.9987	0.691 ± 0.029	0.9727	0.695 ± 0.023	0.9777
*T. cariense*	0.258 ± 0.022	0.9985	0.402 ± 0.041	0.9765	0.291 ± 0.065	0.9911	0.513 ± 0.019	0.9929	0.639 ± 0.038	0.9788	0.847 ± 0.039	0.9633
*T. elegans*	0.344 ± 0.013	0.9776	0.490 ± 0.036	0.9949	0.241 ± 0.003	0.9983	0.417 ± 0.013	0.9861	0.576 ± 0.010	0.9585	0.833 ± 0.056	0.9758

**Table 5:** Half-maximal metal chelating and radical scavenging concentrations (IC_50_, µg/mL) of both water (WE) and methanolic (ME) extracts from *Thlaspi* species.

Antioxidants	DPPH^•^ Scavenging ME		ABTS^•+^ Scavenging ME		DMPD Scavenging ME		Fe^2+^ Chelating ME	
	WE	ME	WE	ME	WE	ME	WE	ME
BHA	15.8	15.8	8.8	15.8	-	15.8	20.4 *	20.4 *
BHA	15.8	15.8	8.8	15.8	-	15.8	20.4 *	20.4 *
BHT	38.5	38.5	9.5	38.5	-	38.5	3.8 **	3.8 **
Trolox	11.7	11.7	8.6	11.7	43.3	11.7	-	-
α-Tocopherol	14.4	14.4	8.9	14.4	-	14.4	-	-
*T. alliaceum*	173.3	-	36.5	36.5	-	346.5	16.9	38.5
*T. arvense*	346.5	-	43.3	77.0	138.6	-	15.4	38.5
*T. violascens*	173.3	-	30.1	40.8	173.3	-	21.0	63.0
*T. aghricum*	231.0	231.0	36.5	43.3	138.6	-	21.0	63.0
*T. cataonicum*	231.0	346.5	38.5	43.3	-	-	19.1	49.5
*T. annuum*	-	-	28.9	28.9	99.0	99.0	30.1	53.2
*T. watsonii*	-	231.0	38.5	38.5	346.5	-	21.0	46.2
*T. cariense*	-	-	69.3	36.5	173.3	-	16.9	15.8
*T. elegans*	-	-	46.2	77.0	99.0	-	26.7	26.1

* These values belong to ascorbic acid, ** These values belong to EDTA.

**Table 6:** The inhibition values (IC_50_, μg/mL) of methanol and water extracts of *Thlaspi* species against carbonic anhydrase isoenzyme II (CA II), acetylcholinesterase (AChE), and α-amylase enzymes.

Antioxidants	hCA II				AChE				α-Amylase			
	Water Extract		Methanol Extract		Water Extract		Methanol Extract		Water Extract		Methanol Extract	
	IC_50_	r^2^	IC_50_	r^2^	IC_50_	r^2^	IC_50_	r^2^	IC_50_	r^2^	IC_50_	r^2^
*T. alliaceum*	-	-	-	-	17.9	0.9479	20.1	0.9549	171.4	0.9091	216.3	0.9183
*T. arvense*	256.5	0.9355	52.0	0.8828	21.9	0.9636	22.6	0.9946	176.8	0.9101	197.8	0.9381
*T. violascens*	-	-	-	-	17.9	0.8988	23.4	0.9813	171.5	0.9249	206.8	0.9151
*T. aghricum*	74.6	0.9687	-	-	23.5	0.9964	21.6	0.9555	171.7	0.9242	188.4	0.9981
*T. cataonicum*	41.9	0.7949	202.0	0.8889	17.3	0.8754	23.2	0.9784	181.9	0.9186	122.4	0.9965
*T. annuum*	60.0	0.7861	-	-	20.0	0.8827	20.8	0.9707	181.1	0.8878	207.5	0.7948
*T. watsonii*	-	-	-	-	20.7	0.9410	21.5	0.9829	168.6	0.9154	176.1	0.8497
*T. cariense*	-	-	-	-	21.5	0.9167	20.7	0.9699	160.2	0.9428	194.7	0.8829
*T. elegans*	-	-	-	-	24.1	0.8791	18.2	0.9065	194.4	0.9285	245.9	0.8787
Acetazolamide *	8.37	0.9825	8.37	0.9825	-	-	-	-	-	-	-	-
Tacrine **	-	-	-	-	5.97	0.9706	5.97	0.9706	-	-	-	-

* Acetazolamide (AZA) is a standard for CA II. ** Tacrine (TAC) is a standard for AChE inhibition.

**Table 7:** *Thlaspi* species and their locations, collection date and collection numbers.

No	Taxa	Location	Collection Date	Collection Number
1	*Thlaspi alliaceum*	Bolu, Abant	21 May 2016	Karaismailoğlu-250
2	*Thlaspi arvense*	Artvin, Borçka	22 May 2015	Karaismailoğlu-167
3	*Thlaspi violascens* *	Osmaniye, Düziçi	26 May 2015	Karaismailoğlu-181
4	*Thlaspi aghricum* *	Ağrı, Hamur	16 May 2015	Karaismailoğlu-162
5	*Thlaspi cataonicum*	Adana, Saimbeyli	18 April 2015	Karaismailoğlu-124
6	*Thlaspi annuum*	Amasya, Taşova	2 May 2015	Karaismailoğlu-143
7	*Thlaspi watsonii* *	Van, Güzeldere	2 July 2015	Karaismailoğlu-210
9	*Thlaspi cariense* *	Muğla, Marmaris	3 April 2015	Karaismailoğlu-121
12	*Thlaspi elegans* *	Niğde, Çamardı	25 May 2015	Karaismailoğlu-173b

*: Endemic taxon.

## Data Availability

The original contributions presented in this study are included in the article. Further inquiries can be directed to the corresponding authors.

## Supplementary Materials

The following supporting information can be downloaded at: https://www.mdpi.com/article/10.3390/plants15142207/s1.
