# Supplementary material for: Integrated Assessment of Bioactive Properties of Nine Thlaspi Species: Antioxidant Activity, Enzyme Inhibition and LC–MS/MS Polyphenolic Characterization
Source: Plants (Basel). 2026 Jul 20;15(14):2207. doi: 10.3390/plants15142207 (PMC13417124; doi:10.3390/plants15142207)
Supplement: Supplementary file 1 [file plants-15-02207-s001.zip › plants-4325888-supplementary.pdf]

SUPPEMENTARY FILE

Figure S1. Reducing abilities of *Thlaspi* species extracts, **A.** Ferric ions (Fe^{3+}) reducing abilities of *Thlaspi* species extracts. **B.** Cupric ions (Cu^{2+}) reducing ability of *Thlaspi* species extracts, **C.** Fe^{3+}-TPTZ complex reducing ability of *Thlaspi* species extracts.

Figure S2. Radical scavenging effects of water and ethanol extracts of *Thlaspi* species. **A**. DPPH$^{\bullet}$ scavenging ability, **B**. ABTS$^{\bullet+}$ scavenging ability, **C**. DMPD$^{\bullet+}$ scavenging ability, **D**. Fe^{2+} chelating ability

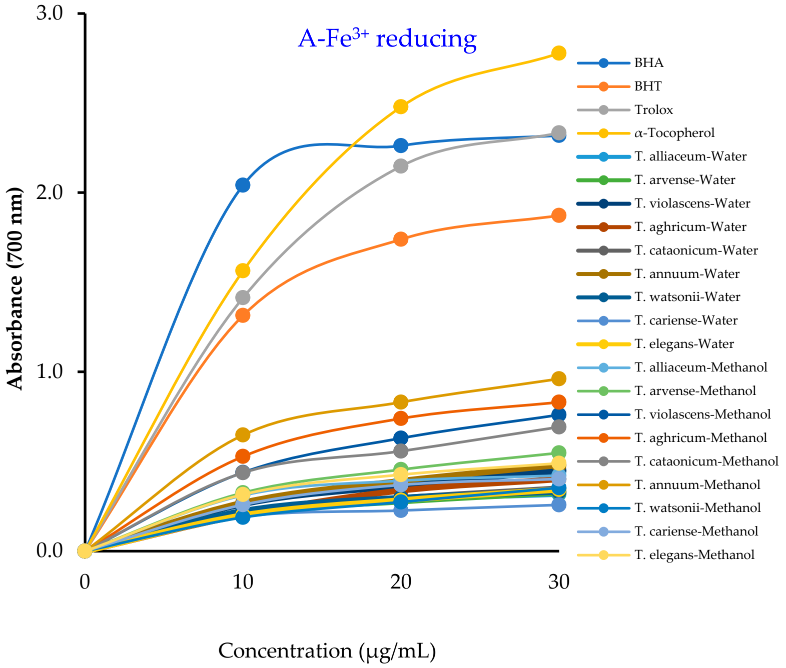

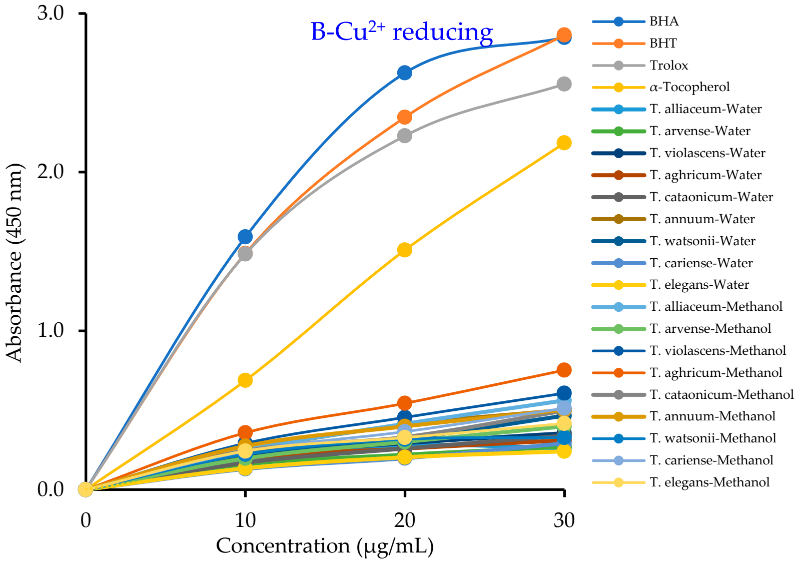

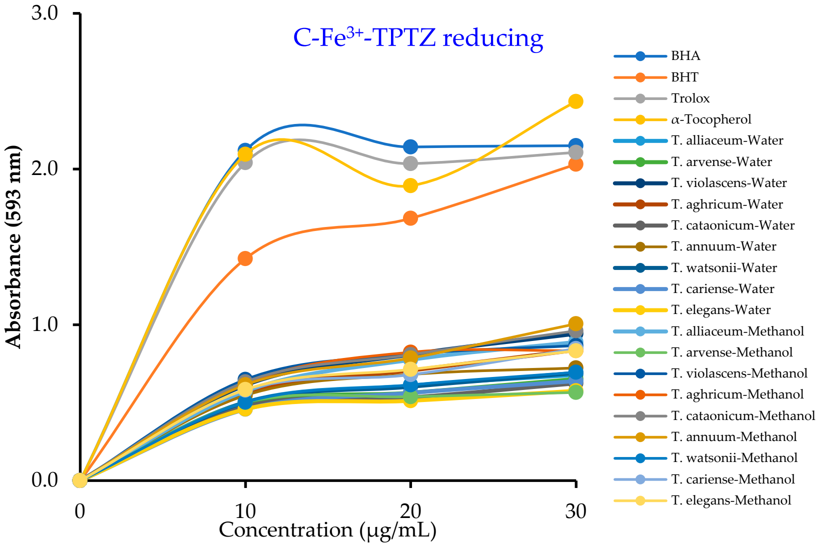

C-Fe3+-TPTZ reducing
Absorbance (593 nm)
3.0
2.0
1.0
0.0
0
10
20
30
Concentration (μg/mL)
BHA
BHT
Trolox
α-Tocopherol
T. alliaceum-Water
T. arvense-Water
T. violascens-Water
T. aghricum-Water
T. cataonicum-Water
T. annuum-Water
T. watsonii-Water
T. cariense-Water
T. elegans-Water
T. alliaceum-Methanol
T. arvense-Methanol
T. violascens-Methanol
T. aghricum-Methanol
T. cataonicum-Methanol
T. annuum-Methanol
T. watsonii-Methanol
T. cariense-Methanol
T. elegans-Methanol

Figure S1

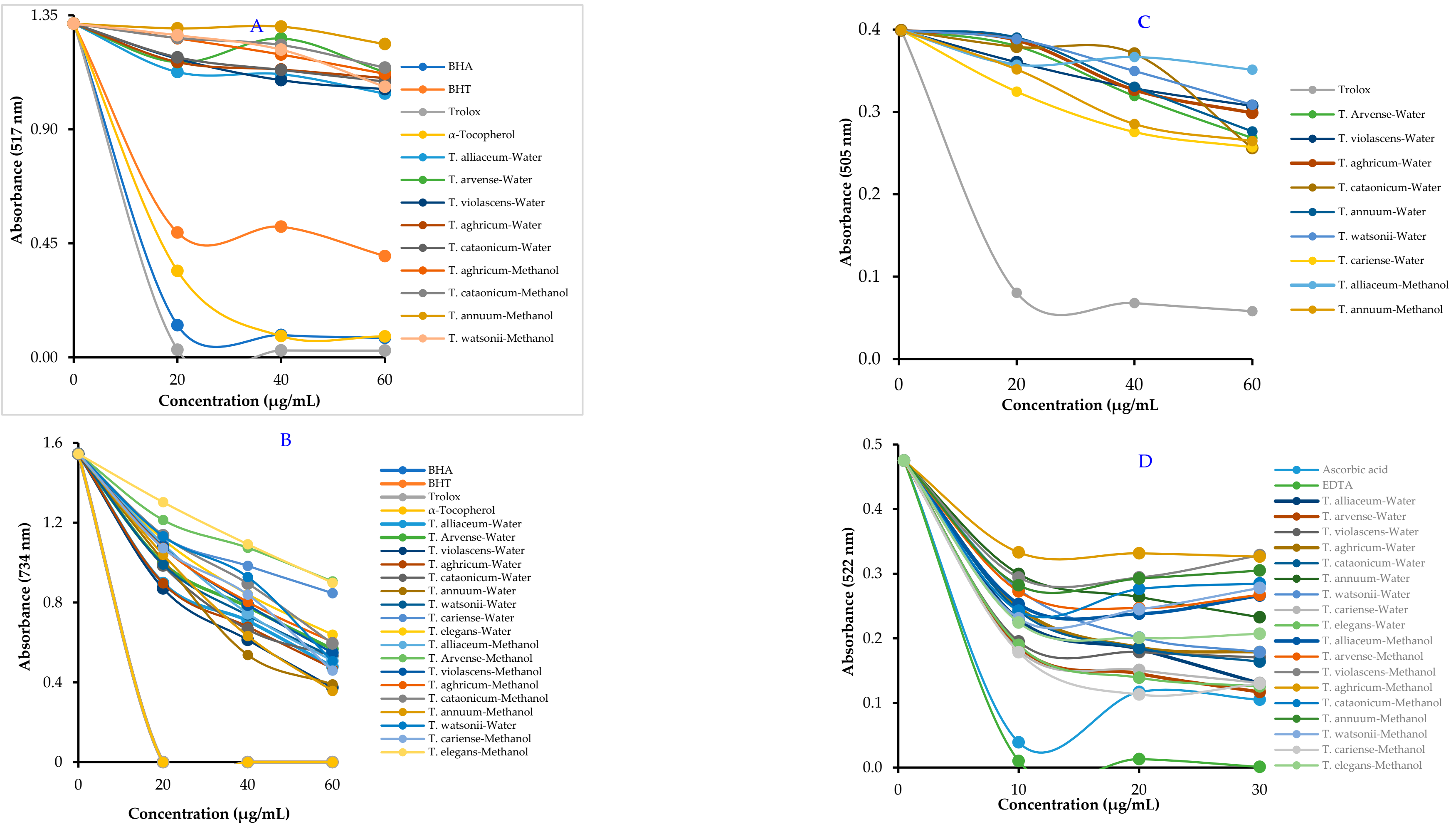
A
Absorbance (517 nm)
Concentration (µg/mL)
1.35
0.90
0.45
0.00
0
20
40
60
BHA
BHT
Trolox
α-Tocopherol
T. alliaceum-Water
T. arvense-Water
T. violascens-Water
T. aghricum-Water
T. cataonicum-Water
T. aghricum-Methanol
T. cataonicum-Methanol
T. annuum-Methanol
T. watsonii-Methanol
C
Absorbance (505 nm)
Concentration (µg/mL
0.4
0.3
0.2
0.1
0.0
Trolox
T. Arvense-Water
T. violascens-Water
T. aghricum-Water
T. cataonicum-Water
T. annuum-Water
T. watsonii-Water
T. cariense-Water
T. alliaceum-Methanol
T. annuum-Methanol
B
Absorbance (734 nm)
Concentration (µg/mL)
1.6
1.2
0.8
0.4
0
BHA
BHT
Trolox
α-Tocopherol
T. alliaceum-Water
T. Arvense-Water
T. violascens-Water
T. aghricum-Water
T. cataonicum-Water
T. annuum-Water
T. watsonii-Water
T. cariense-Water
T. elegans-Water
T. alliaceum-Methanol
T. Arvense-Methanol
T. violascens-Methanol
T. aghricum-Methanol
T. cataonicum-Methanol
T. annuum-Methanol
T. watsonii-Water
T. cariense-Methanol
T. elegans-Methanol
D
Absorbance (522 nm)
Concentration (µg/mL)
0.5
0.4
0.3
0.2
0.1
0.0
10
30
Ascorbic acid
EDTA
T. alliaceum-Water
T. arvense-Water
T. violascens-Water
T. aghricum-Water
T. cataonicum-Water
T. annuum-Water
T. watsonii-Water
T. cariense-Water
T. elegans-Water
T. alliaceum-Methanol
T. arvense-Methanol
T. violascens-Methanol
T. aghricum-Methanol
T. cataonicum-Methanol
T. annuum-Methanol
T. watsonii-Methanol
T. cariense-Methanol
T. elegans-Methanol

Figure S2
